# Comparison of dysfunctional attitudes, cognitive vulnerability to depression, before and during the COVID-19 pandemic in healthy participants

**DOI:** 10.1186/s40359-024-01674-0

**Published:** 2024-04-04

**Authors:** Haruka Muraosa, Toshinori Shirata, Yusuke Saito, Keisuke Noto, Akihito Suzuki

**Affiliations:** Department of Psychiatry, Yamagata University School of Medicine, 2-2-2 Iida-Nishi, Yamagata city, 990-9585 Japan

**Keywords:** COVID-19, DAS-24, Dependency, Dysfunctional attitude

## Abstract

**Background:**

During the COVID-19 pandemic, depression and suicide rates increased worldwide, and in Japan. Presumably, an increase of neuroticism-related personality traits mediates the relation linking the COVID-19 pandemic with depression and suicide. This study examined COVID-19 pandemic effects on dysfunctional attitudes, cognitive vulnerability to depression, in healthy participants.

**Methods:**

The study used Dysfunctional Attitude Scale (DAS) -24 data of three subscales (i.e., achievement, dependency, and self-control) obtained from 270 Japanese medical students during October 2017 – June 2022. Participants were divided into two groups: those for whom DAS-24 was assessed before the pandemic (phase 1 group, October 2017 – March 2020, *n* = 178) and those for whom DAS-24 was assessed during the pandemic (phase 2 group, April 2020 – June 2022, *n* = 92).

**Results:**

Total DAS-24 scores of the phase 2 group were significantly (*p* = 0.047) lower than those of the phase 1 group. Scores of the dependency subscale for the phase 2 group were significantly (*p* = 0.002) lower than those for the phase 1 group, but no significant difference was found in the scores of the achievement and self-control subscales.

**Conclusions:**

These findings suggest that a decrease in DAS-24 scores, particularly of the dependency subscale, occurred during the COVID-19 pandemic. Possible mechanisms underlying these results are 1) individuals became less preoccupied with receiving evaluation, 2) individuals realized that self-cognition depending on the approval of others is unimportant, and 3) high levels of dysfunctional attitude were maladaptive for obtaining affective benefits via social interactions during the COVID-19 pandemic.

**Supplementary Information:**

The online version contains supplementary material available at 10.1186/s40359-024-01674-0.

## Introduction

In November 2019, COVID-19 infected people in Wuhan, China. It had spread quickly worldwide by March 2020. The COVID-19 pandemic strongly affected global public health. Many changes were recorded in relation to mental health, economic and social restrictions, decreased interpersonal interaction, and longer hours spent at home. Depression and suicide increased around the world and in Japan [1–3]. Because depression and suicide are related to neuroticism-related personality [4–6] and because neuroticism-related personality is exacerbated by negative life events [7], an increase in neuroticism-related personality trait presumably mediated the relation of the COVID-19 pandemic with increased depression and suicide. Earlier studies have examined COVID-19 pandemic effects on personality traits [8, 9]. Sutin et al. [8] conducted an online survey from the beginning of February 2020 to mid-March 2020, with participants from across the United States representing various age groups. Results indicated a reduction in neuroticism during the COVID-19 pandemic. Krautter et al. [9] also inferred a decline in neuroticism during the COVID-19 pandemic among first-year German university students. These results are rather unexpected and contrary to the prevailing hypothesis, necessitating replication studies particularly addressing other neuroticism-related personality traits associated with vulnerability to depression and suicide.

According to Beck's cognitive theory, depression results from maladaptive beliefs formed by negative childhood experiences. Once these maladaptive self-schemas are activated by life stressors, these beliefs engender cognitive errors and negative thoughts, resulting in negative interpretations and self-evaluations that characterize clinical depression [10, 11]. Weissman and Beck [12] developed the Dysfunctional Attitude Scale (DAS) to measure these maladaptive beliefs. Power et al. [13] conducted a factor analysis of the DAS items and classified dysfunctional attitudes into three groups. They found that depression-prone individuals had dysfunctional attitudes, not in all areas of their lives, but in one or two highly valued areas. An empirical study by Sun et al. [14] indicated that negative life events can increase the DAS scores of college students with major depressive disorders.

Specifically considering the relation among negative life events, dysfunctional attitudes, and depression, it was hypothesized that DAS scores increased during the COVID-19 pandemic.

## Methods

This study was conducted as a part of studies examining the association among parental attitudes, genetic factors, and personality [15, 16]. Data from October 2017 – June 2022 were used for this study. All 270 participants were students at the Yamagata University School of Medicine. No participant was married. Among the participants, 19 were cohabitating with family members; all others were living alone. Also, 3 participants were current smokers; 66 reported drinking alcohol regularly. For this study, the exclusion criteria were 1) having a severe physical disease and 2) having a prior or current psychiatric disorder. The 140 male participants and 130 female participants were of mean ± SD (range) age of 23.3 ± 1.6 years (21–29). The study protocol was approved by the Ethics Committee of Yamagata University School of Medicine. All participants provided written informed consent to participate.

Power et al. [13] developed a 24-item version of the DAS (DAS-24) with three subscales: achievement, dependency, and self-control. The achievement subscale includes items related to achievement and failure. The dependency subscale includes items related to dependence on evaluation and approval from others. The self-control subscale includes topics related to self-control. The achievement subscale consists of eight items related to achievement and failure, such as “If I fail partly, it is as bad as being a complete failure.” The dependency subscale consists of eight items, such as “I am nothing if a person I love does not love me.” The self-control subscale consists of eight items about the need for self-control, such as “A person should do well at everything he undertakes.” The Japanese version of the DAS-24, which has been shown to have high reliability and validity [17], was used for this study.

The Japanese government declared the first state of emergency for COVID-19 in April 2020 and requested that Japanese residents refrain from going out of their homes for non-essential reasons [18]. Therefore, we divided the participants into two groups: those for whom the DAS-24 was administered before the COVID-19 pandemic, the phase 1 group (October 2017 – March 2020; *n* = 178); and those for whom it was administered during the COVID-19 pandemic, the phase 2 group (April 2020 – June 2022, *n* = 92).

For the present sample, the Shapiro–Wilk test results indicated that the self-control subscale scores did not follow a normal distribution (*p* = 0.017), although the scores of the other DAS-24 scores were normally distributed. Consequently, statistical analyses were conducted using Student *t*-tests or Pearson correlation analyses for the total DAS-24 scores and the scores of achievement and dependency subscales. Mann–Whitney U tests or Spearman correlation analyses were used for scores of the self-control subscale. All statistical analyses were conducted using software (SPSS 26; SPSS Japan Inc.). A *p* value of less than 0.05 was inferred as significant.

## Results

In this sample, mean age (mean ± SD; 23.3 ± 1.7 in the phase 1 group vs. 23.3 ± 1.5 in the phase 2 group; *t* = 0.126, *p* = 0.900) and sex ratio (male/female; 92/86 in the phase 1 group vs. 48/44 in the phase 2 group; χ^2^ = 0.006, *p* = 0.939) were not different between the phase 1 group and the phase 2 group.

The associations of age, sex, presence of cohabitants, and drinking habits are presented in Supplementary Table 1 along with the DAS-24 total and three subscale scores. No significant association was found between these factors and the DAS-24 scores (Suppl. Table 1).

**Fig. 1:**
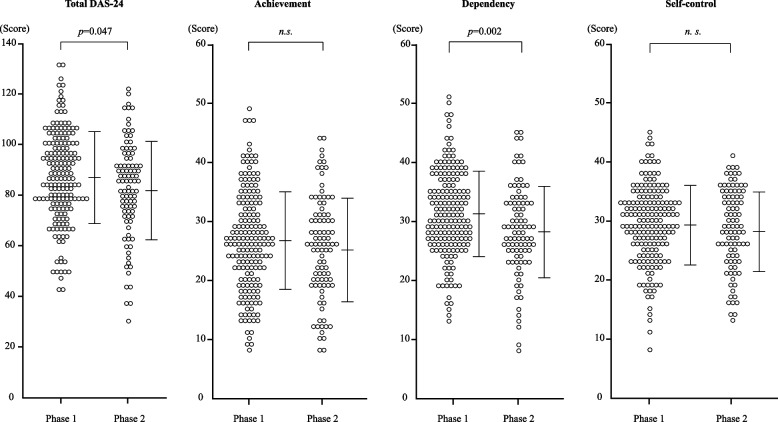
Comparison of DAS-24 total and three subscale scores between phase 1 and phase 2 groups. *P* values represent results of Student *t*-tests or Mann–Whitney U tests. DAS-24 and n.s. respectively denote the 24-item Dysfunctional Attitude Scale and not significant. Bracketed bars represent SD. Horizontal lines in the middle show means

Table 1 and Fig. 1 present the DAS-24 total and the three subscale scores for the phase 1 and phase 2 groups.

**Table 1:** DAS-24 total and three subscale scores in phase 1 and phase 2 groups

	Phase 1 group	Phase 2 group	*t* or *U*	*P*
	(*n* = 178)	(*n* = 92)		
DAS-24 (SD)				
Total	87.2 (18.1)	82.4 (19.4)	1.998	0.047
Achievement	26.6 (8.5)	25.4 (8.7)	1.036	0.301
Dependency	31.3 (7.3)	28.3 (7.7)	3.079	0.002
Self-control	29.3 (6.5)	28.6 (6.9)	7860.5	0.59

Phase 1 group and phase 2 groups respectively include participants for whom the DAS-24 was assessed before the pandemic (October 2017 – March 2020) and during the pandemic (April 2020 – June 2022)

*P* values represent results of Student t-tests for the scores of total DAS-24 and the scores of achievement and dependency subscales or Mann–Whitney U test for the scores of self-control subscale. DAS-24 denotes the 24-item Dysfunctional Attitude Scale

The total DAS-24 scores were significantly (*p* = 0.047) lower for the phase 2 group than for the phase 1 group (Table 1 and Fig. 1). The dependency subscale scores were significantly (*p* = 0.002) lower for the phase 2 group than for the phase 1 group. No significant difference was found in the scores of the achievement subscale (*p* = 0.301) or the self-control subscale (*p* = 0.590) (Table 1 and Fig. 1).

## Discussion

Contrary to our hypothesis, results of this study demonstrated that the DAS-24 total and dependency subscale scores were significantly lower in the phase 2 group (participating during the COVID-19 pandemic) than in the phase 1 group (participating before the COVID-19 pandemic). These findings are in line with results obtained from results of earlier studies suggesting that neuroticism, which is positively correlated with the DAS-24 scores [19], decreased during the COVID-19 pandemic [8, 9, 20].

The exact mechanism underlying these findings remains unclear. However, one plausible mechanism is a decrease in the frequency of direct evaluations by others. During the COVID-19 pandemic, the Japanese Government restricted public gatherings. Online classes for students and remote work for employees were enforced for a long time [21]. Decreased direct interpersonal interactions because of the COVID-19 pandemic might have caused individuals to become less preoccupied with receiving evaluations and approval from external sources, consequently leading to reduction of DAS-24 scores, and especially of the dependency subscale scores. East Asians, including Japanese people, have a stronger tendency, compared to North American and European people, to be concerned about gaining the approval of community members [22]. Consequently, the DAS-24 score reduction might be exaggerated in this sample, which consists entirely of Japanese people.

Another possible explanation is a change in the participants' cognition. In the maladaptive belief that life is worthless and unfulfilling without the approval of others, an unconscious assumption is that approval from others is reliable and beneficial for self-protection. During the COVID-19 pandemic, however, prevailing social attitudes changed: active interaction with numerous people was considered irresponsible, whereas remaining at home and socializing only with family were regarded as duties of sensible good citizenship. Traveling and cultural activities with others were judged as “unnecessary”. People might have learned that approval from others and the values they share with others are unstable. They might have come to believe that no definitive safety exists in life, irrespective of how much approval they receive from others. Therefore, self-cognition depending on the approval of others might have been altered because of the COVID-19 pandemic.

Alternatively, individuals who engage in a greater number of social interactions are widely recognized as experiencing affective benefits such as increased happiness and less sadness, compared to people who participate in fewer social interactions [23]. Monninger et al. [24] investigated the affective benefit from social interactions in relation to neuroticism using a longitudinal birth cohort, and demonstrated that individuals with higher neuroticism exhibited the affective benefit from more social interactions before the COVID-19 pandemic. By contrast, during the pandemic, the mood-lifting effects of social interactions were found only in individuals with lower neuroticism [24]. Those findings suggest that high levels of neuroticism are maladaptive on social affective gain during the pandemic [24]. Given the positive correlation between the DAS-24 scores and neuroticism [19], the results of this study might be explained by adaptation of personality to the COVID-19 pandemic, wherein the decreases in the DAS scores during the pandemic were associated with increased probability for obtaining affective benefits from social interactions.

There are several limitations of this study. First, our participants were all young, Japanese, highly educated medical students who had much information about infectious diseases. A longitudinal study by Daly et al. [25] showed that increases in the prevalence of mental health problems during the pandemic were pronounced in young and highly educated groups. Furthermore, changes in personality caused by the pandemic were reportedly moderated by age and ethnicity [20]. Therefore, generalizing the current findings to broader populations or other ethnic groups might be difficult. Second, the sample size examined for this study was small. Third, this study used a cross-sectional design with a limited time frame. From results of a longitudinal study, Sutin et al. [20] reported that neuroticism declined early during the COVID-19 pandemic, but changes in neuroticism later in the pandemic led to reversion to the state which had prevailed before the pandemic. Consequently, this study should be followed up with a longitudinal study to ascertain whether the COVID-19 pandemic causes a permanent change in dysfunctional attitudes. Fourth, we did not assess social interactions, purpose in life, stress mindset, positivity, or a silver lining, any of which can be a mediator between the pandemic and DAS-24 scores [24, 26–28].

## Conclusions

The findings obtained from this study suggest that the DAS-24 total and dependency subscale scores decreased during the COVID-19 pandemic. Possible mechanisms underlying these results are 1) individuals became less preoccupied with receiving evaluation, 2) individuals realized that self-cognition depending on the approval of others is unimportant, and 3) high levels of dysfunctional attitude were maladaptive for obtaining the affective benefits via social interactions during the COVID-19 pandemic.

### Supplementary Information

**Supplementary Material 1. **

## Data Availability

No datasets were generated or analysed during the current study.
